# Giant Cell Tumor of the Distal Radius: Wide Resection, Ulna Translocation With Wrist Arthrodesis

**DOI:** 10.7759/cureus.15034

**Published:** 2021-05-15

**Authors:** Alok C Agrawal, Ankit Kumar Garg, Ranjeet Choudhary, Shilp Verma, Rudra Narayan Dash

**Affiliations:** 1 Orthopaedics, All India Institute of Medical Sciences (AIIMS) Raipur, Raipur, IND; 2 Orthopaedics, Ganga Medical Centre & Hospitals, Coimbatore, IND; 3 Orthopaedic Surgery, All India Institute of Medical Sciences (AIIMS) Raipur, Raipur, IND

**Keywords:** giant cell tumour, distal radius, wide resection, ulna translocation, wrist arthrodesis

## Abstract

Giant cell tumor (GCT) of the bone is a locally aggressive neoplasm and usually managed with extended curettage and adjuvant therapy, which is associated with reduced risk of recurrence. The juxta-articular distal radius giant cell tumor is challenging due to the destruction of subchondral bone and articular cartilage, making it difficult to salvage the wrist joint anatomy and function. Various methods described include wide resection and reconstruction of allograft or centralization of the ulna with wrist arthrodesis. We present the functional outcome of distal end radius GCT, which was successfully managed with wide local excision, ulna translocation, and wrist arthrodesis. At the two years follow-up, the patient shows excellent functional outcome with supination and pronation movements and no local recurrence.

## Introduction

Giant cell tumor (GCT) of the bone is a solitary, benign, but locally aggressive neoplasm. It commonly involves the epiphyseal region of long bones in the mature skeleton. It predominantly occurs in the distal femur, proximal tibia, and distal radius [1]. Distal radius GCT accounts for approximately 10% of total skeletal GCT [2]. As per the current concepts for surgery of GCT, the aim is to salvage normal anatomy after complete removal of tumor, prevent its recurrence, and restore forearm and hand function to as normal as possible. The standard treatment of GCT of the distal radius is extended curettage and reconstruction using bone grafts or polymethylmethacrylate (PMMA) cementation. This surgery is associated with recurrence even after adjuvant therapy like phenol, liquid nitrogen, etc. Local recurrence as high as 5-17% is usually observed in the first two years of surgery [3,4]. Microscopic tumor residuals cause recurrence of GCT following extended curettage.

The juxta-articular distal radius GCT is challenging when associated with the destruction of subchondral bone and articular cartilage, making it difficult to salvage the wrist joint anatomy and function. Various methods described include wide resection and reconstruction using structural autografts fibula (vascularized/non-vascularized), allograft, or centralization of the ulna with wrist arthrodesis and are reported with lower recurrence rate [5,6]. Reconstruction of distal radius by ipsilateral proximal fibular arthroplasty although practiced commonly is associated with disadvantages such as non-anatomical reconstruction, non-union, and donor site morbidity. Seradge described a novel technique of distal radius reconstruction by ulnar translocation along with its soft tissues [7]. It has an added advantage of having a local vascularised graft without the need of microsurgical procedures but can lead to weaker grip strength and loss of movements at the wrist [2].

We present a case of distal radius GCT managed successfully with wide resection, translocation of the ulna, and wrist arthrodesis to preserve pronation and supination of forearm and hand function with excellent functional outcome.

## Case presentation

**Figure 1:**
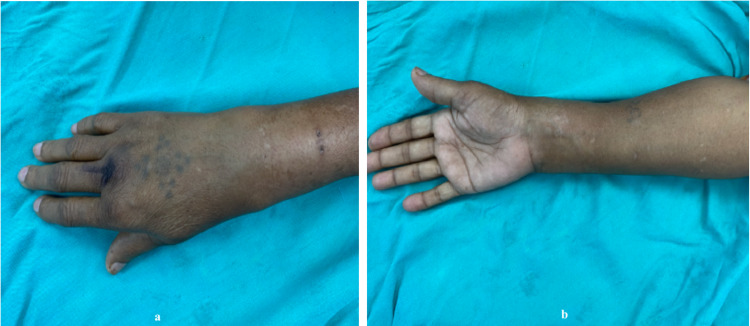
(a & b) Showing clinical photograph of the right forearm and wrist with giant cell tumor of the distal radius.

A 57-year-old right hand dominant female, presented to us with pain and swelling at the right wrist for six months. On examination, there was a non-tender, solitary, firm swelling at distal radius measuring approximately 5 x 3 cm in size, with mobile overlying skin and dilated veins. The swelling was non-fluctuant, and the local temperature was mildly raised. There was no distal neurovascular deficit, and hand functions were normal (Figure 1).

**Figure 2:**
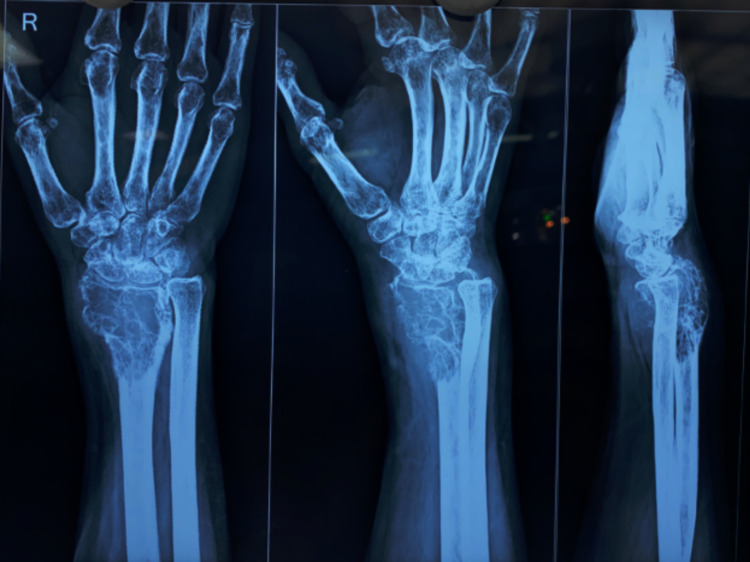
Pre-operative radiograph of right wrist showing locally aggressive giant cell tumor (GCT) of the right distal radius.

**Figure 3:**
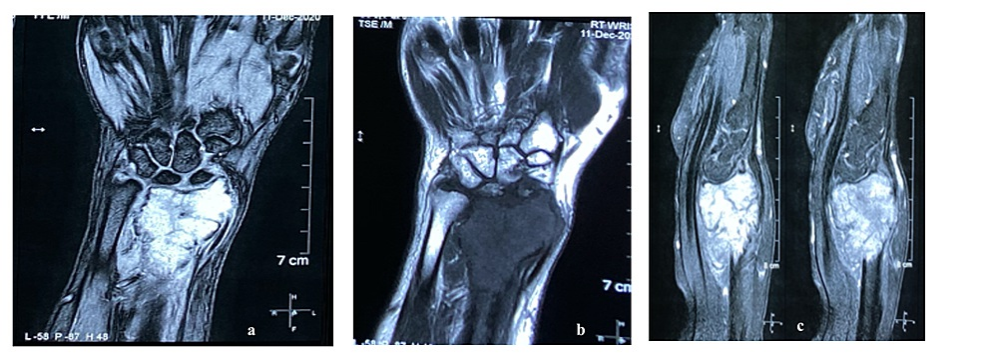
(a & c) MRI T2 image showing lesion at distal radius and extra compartmental involvement, and (b) MRI T1 image showing the extent of the lesion.

Radiological evaluation with X-ray (Figure 2) revealed an aggressive, eccentric, expansile, osteolytic lesion present at epiphysis extending to metaphysis in the right distal radius, approximately measuring around 6 x 3 cm. The transition zone was narrow, with a cortical breach in the metaphyseal region and subchondral bone destruction. However, there was a minimal periosteal reaction. The aforementioned radiological features suggest a GCT of the distal radius, which was later confirmed on core needle biopsy. An MRI scan of the distal radius was done to assess the soft tissue involvement and medullary extension of the tumor. The scan showed involvement of distal radius articular margin with soft tissue extension (Figure 3).

**Figure 4:**
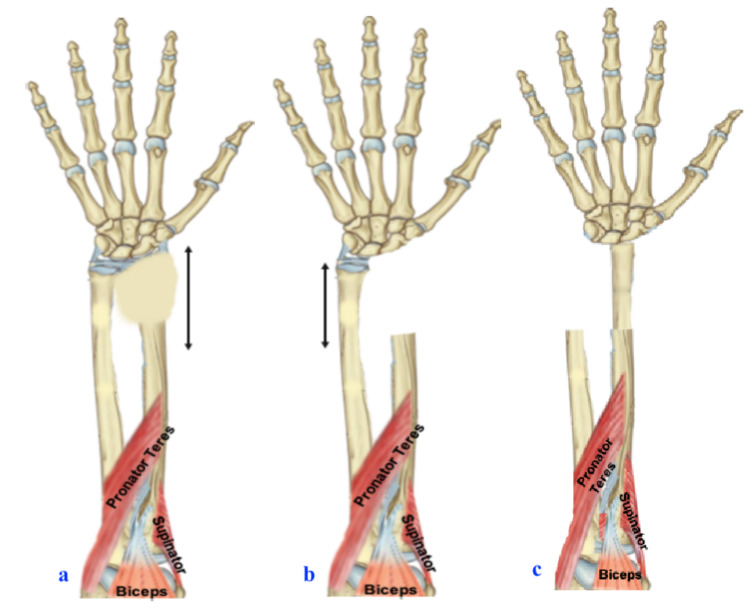
(a) Plan for wide resection of distal radius. (b) Distal ulna osteotomy. (c) Translocation of distal ulna on radius cut end.

**Figure 5:**
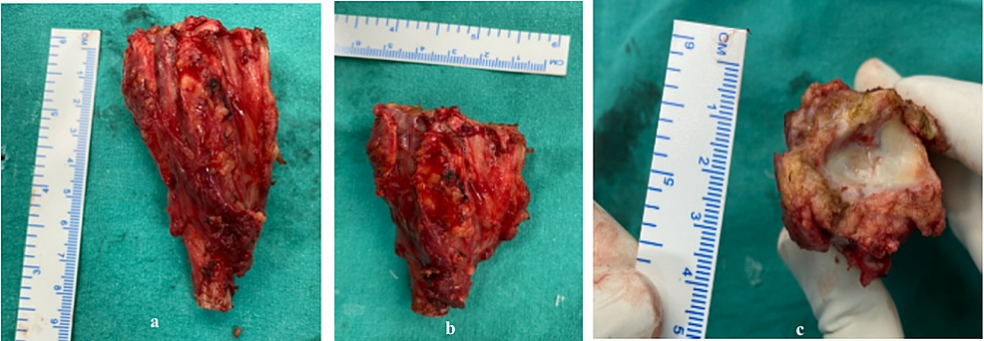
(a-c) Specimen showing excised wide resection of distal radius in three dimension.

The patient was planned for wide resection, ulna translocation, and wrist arthrodesis (Figure 4 shows detailed planning). Level of radius resection was planned according to MR finding, including 1 cm of normal healthy uninvolved bone. Under general anesthesia, through a dorsal approach centering over the third metacarpal, the tumor was exposed. Around 8 cm of the distal radius was excised along with pronator quadratus through radio-carpal, and the radio-ulnar joint capsule (Figure 5).

**Figure 6:**
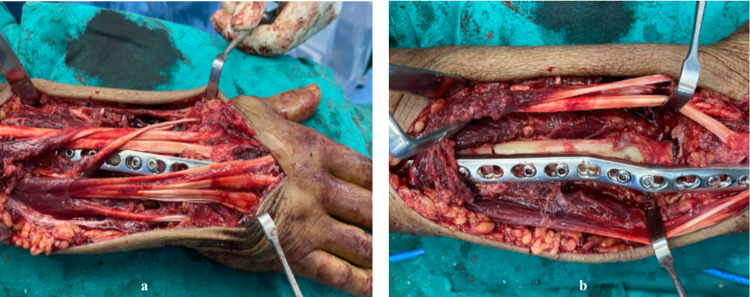
Intra-operative images, final fixation with 3.5 mm pre-contoured locking plates.

**Figure 7:**
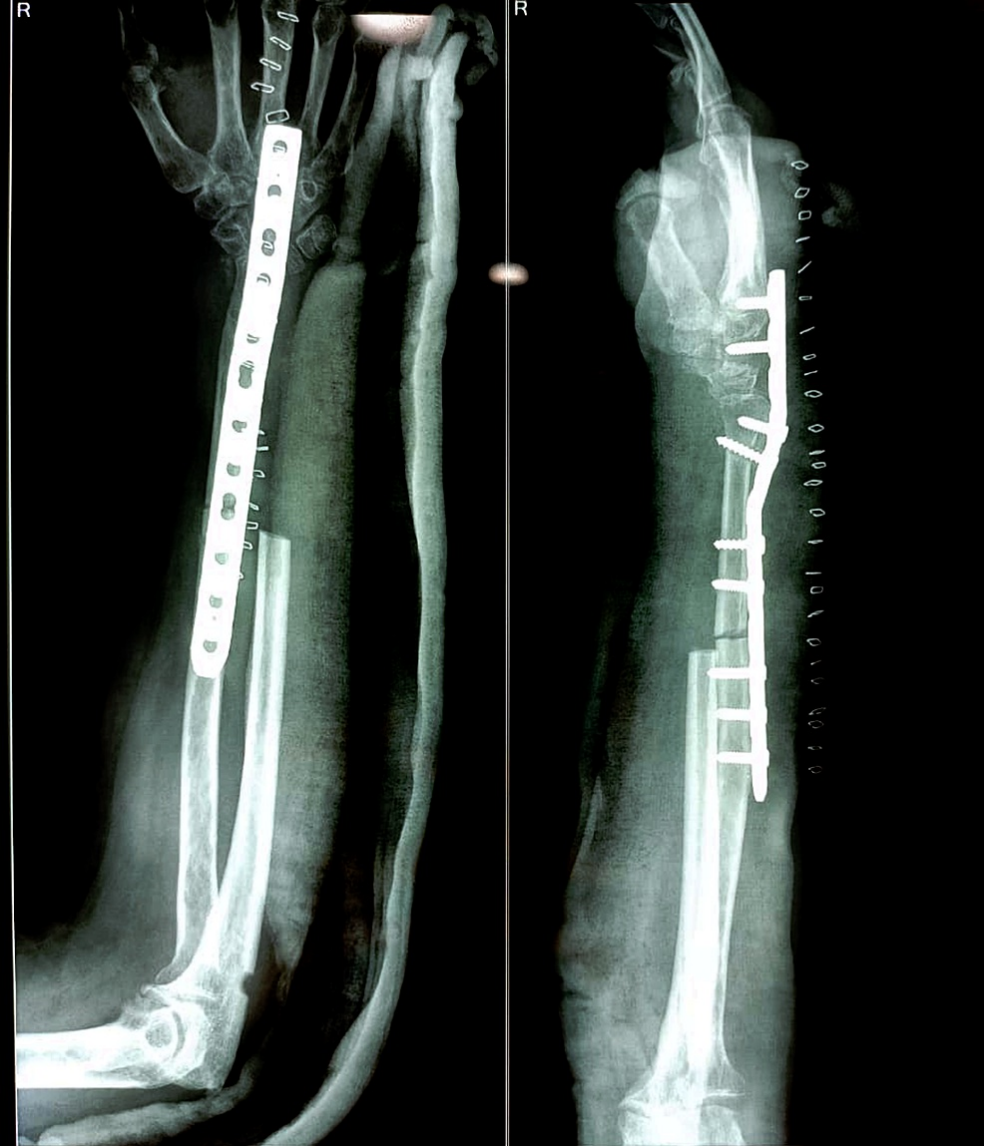
Immediate post-operative radiograph.

The tumor bed was treated with a 3% hydrogen peroxide solution to sanitize the wound for microscopic tumor cell spillage. Around 8 cm of the distal ulna was resected, keeping intact all soft tissue attachments, and centralized between the lunate column and the radius's cut end. Articular surfaces of distal ulna and lunate were removed with burr for arthrodesis, and it was stabilized with a pre-contoured 3.5 mm locking plate across the wrist over the 3rd metacarpal (Figures 6-7). The wound was closed in layered manner over the negative suction drain to prevent hematoma formation, which was removed after 48 hours. A fixed above elbow slab was applied for two weeks. The incision healed with primary intention, and stitches were removed after two weeks. She was advised to wear removable wrist splint and do intermittent physiotherapy (supination/pronation, finger movements, and sponge ball therapy) exercises. The splint was discontinued after radiological signs of union.

**Figure 8:**
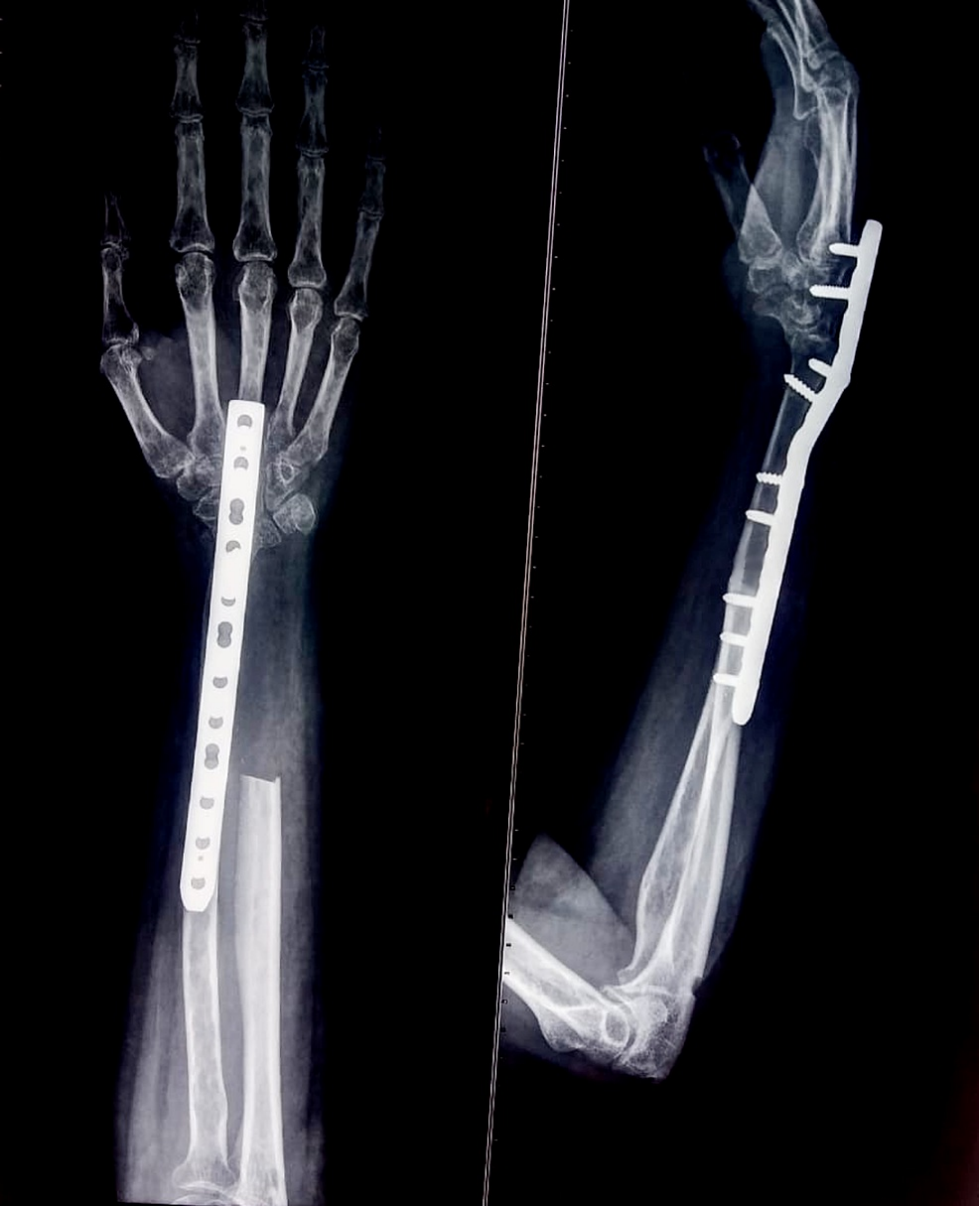
Two-year follow-up radiograph shows bony union at the radio-ulnar junction with no sign of recurrence.

**Figure 9:**
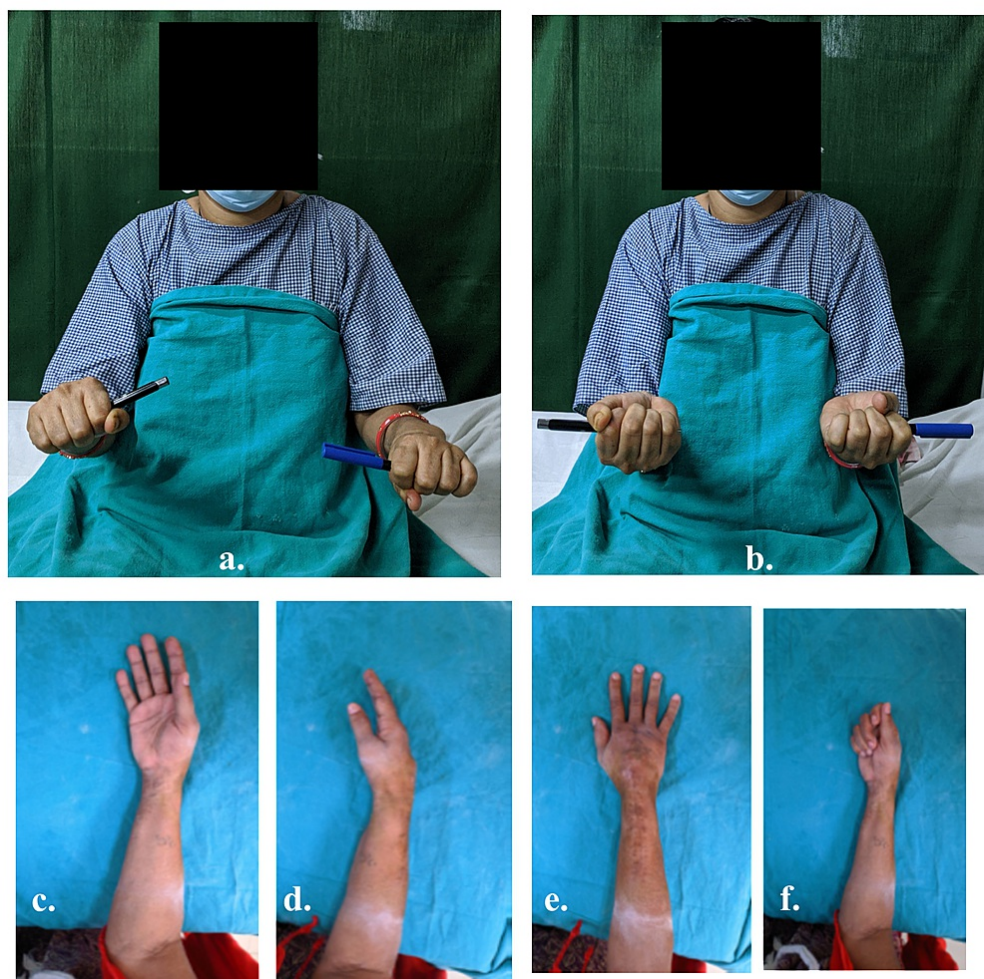
Demonstrating supination and pronation of forearm at final follow-up.

Post-operatively patient was followed up for two years with no clinical and radiological sign of recurrence (Figure 8). At the final follow-up, we assessed the functional outcome with Musculoskeletal Tumor Society (MSTS) scoring system [8]. It is based on patient-specific factors (pain, functional activities, emotional acceptance) and upper limb specific factors (hand position, lifting ability, manual dexterity). The patient achieved a range of 70 degrees of supination and 80 degrees of pronation (Figure 9), good functional finger grip, and MSTS score 28/30.

## Discussion

GCT is a locally aggressive tumor, and an intralesional excision in the form of curettage is associated with a high rate of recurrence [4]. Few case reports state that GCT of the distal radius is unpredictable, aggressive, and often metastasizes to distant organs, most commonly to lungs [9]. The prospect of surgical excision and post reconstruction functional deficiency should be kept in mind, while planning wide local excision to prevent the local recurrence. Many patients who underwent intralesional extended curettage without adequate residual subchondral bone support had secondary joint collapse and degenerative osteoarthritis along with recurrence [10].

Various treatment options available include En-Block resection of the lesion and reconstruction with ipsilateral proximal fibula autograft (vascularized/non-vascularized), tri-cortical iliac graft, structural allografts, distal ulnar centralization, etc. [5]. Reconstruction with non-vascularised fibular autograft has been used by various authors with successful results [6]. In these cases, problems such as non-union (12-38%), fracture of graft (13-29%) and risk of infection are not uncommon [11]. The increased operative time and additional comorbidity to the donor area are other limitations. The need for advanced microsurgical techniques often limits the use of vascularized grafts in such scenarios. Allografts are readily available only in advanced orthopaedic setups [11].

**Figure 10:**
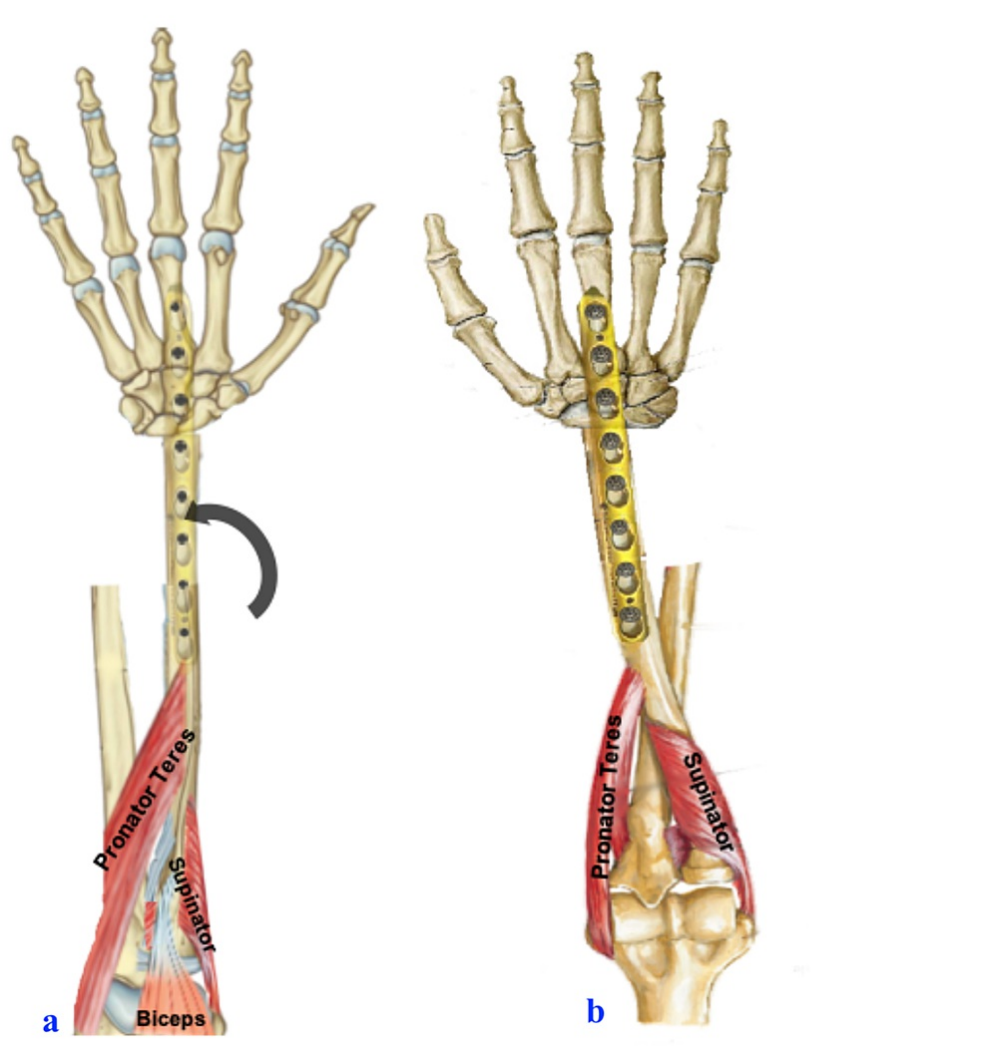
Illustration of forearm showing supination and pronation rotation movements with final fixation.

Following distal radial resection, ulnar translocation to radius and wrist arthrodesis is a shorter duration procedure that utilizes a single incision and local availability of distal ulnar vascularized graft. The technique was described by Seradge but only a few cases have been reported [2,7,12-16]. With the minimal dissection of the ulna soft tissue attachments and preserved muscle attachment [Flexor carpi ulnaris (FCU) was retained], the vascularity of the ulna graft is maintained, which helps in the early bone union, decreases infection rates, and maintains good finger grip strength [2]. Even though we have created a one-bone forearm, the preservation of pronator teres attachment in the ulna helps in pronation, and biceps along with the supinator acting as a forearm supinator. Hence, this procedure conserves rotation (Figure 10 shows graphical representation).

Our results are comparable with other authors as compared in Table 1.

**Table 1:** Review of cases reported in literature with ulnar translocation and wrist arthrodesis after wide excision of giant cell tumor (GCT) distal radius.

Author	No of Cases	Outcome
Seradge, 1982 [7]	2	85% forearm rotations
Lalla and Bhupathi, 1987 [12]	1	Nearly full rotations
Bhan and Biyani, 1990 [13]	6	Supination – 68.3 Degree, Pronation – 73.3 Degree, 62.5% Grip Strength
Chalidis and Dimitriou, 2008 [15]	1	Optimum outcome, no recurrence
Puri et al., 2010 [16]	12	Good outcome in 11 complications Radioulnar synostosis 1 Recurrence 3
McLean et al., 2014 [17]	3	Good forearm rotation -2, One patient with loss of supination
Salunke et al., 2017 [2]	22 (Grade 3)	Good outcome Complications Recurrence 1, Non-Union 1, Graft Fracture 1, Surgical site infection 1

The tumor’s local recurrence is not dependent on the reconstruction technique, but meticulous en bloc excision without tumor spillage in the wound. The translocated distal ulna acts as a vascularized graft in this surgery. The theoretical complication of protrusion of the proximal ulna cut end during supination and pronation has not been observed by us as well as by most of the authors [2,7,16]. Radio-ulnar synostosis could be a potential complication, if periosteal sleeve of translocated ulna maintains a continuity with native ulnar periosteum. One such case of radio-ulnar synostosis was reported by Puri et al. [16]. Our patient did not notice any complications and local recurrence till the last follow-up, and the patient has a considerably good functional outcome (MSTS score -28/30).

## Conclusions

Ulnar translocation and wrist arthrodesis is a simple method of wrist reconstruction with local availability of vascularised graft and provides excellent forearm function and strength.
